# Hymenopteran parasitoid complex and fall armyworm: a case study in eastern India

**DOI:** 10.1038/s41598-024-54342-z

**Published:** 2024-02-18

**Authors:** Subhajit Pal, Swarnali Bhattacharya, Tapamay Dhar, Ankita Gupta, Arunava Ghosh, Sandip Debnath, Nikhitha Gangavarapu, Prajna Pati, Nilanjana Chaudhuri, Hirak Chatterjee, Sabita Kumar Senapati, Prateek Madhab Bhattacharya, Mahesh Kumar Gathala, Alison M. Laing

**Affiliations:** 1 Visva-Bharati University, Santiniketan, Birbhum, West Bengal 731235 India; 2 Uttar Banga Krishi Viswavidyalaya (UBKV), Pundibari, Coochbehar, West Bengal 736165 India; 3 ICAR-National Bureau of Agricultural Insect Resources (NBAIR), Bengaluru, Karnataka 560024 India; 4 University of Nebraska-Lincoln, Lincoln, NE 68583-0816 USA; 5 Siksha ‘O’ Anusandhan Deemed to be University, Bhubaneswar, Odisha 751030 India; 6 International Maize and Wheat Improvement Center (CIMMYT), Dhaka, 1212 Bangladesh

**Keywords:** Plant sciences, Zoology

## Abstract

Fall armyworm (FAW), *Spodoptera frugiperda* (J.E. Smith) has significantly affected maize crop yields, production efficiency, and farmers’ incomes in the Indian Eastern Gangetic Plains region since it was first observed in India in 2018. A lack of awareness by maize growers of the appropriate selection, method, and timing of insecticide application not only creates a barrier to sustainable FAW control but also contributes to increased environmental pollution, reduced human health and increased production costs. We demonstrated that FAW inflicted the most damage in early whorl growth stage of maize, regardless of whether chemical insecticides were applied. FAW egg masses and larvae collected from maize fields in which no insecticides had been sprayed showed high parasitism rates by parasitoid wasps; in contrast fields that had been sprayed had much lower rates of parasitism on FAW. Ten hymenopteran parasitoids were observed in maize fields across the study region, suggesting a diversity of natural methods to suppress FAW in maize at different growth stages. These included two FAW egg parasitoids and eight FAW larval parasitoids. *Microplitis manilae* Ashmead was the most abundant FAW larval parasitoid species, and *Telenomus* cf. *remus* was the dominant FAW egg parasitoid species. Endemic FAW parasitoids such as those observed in this study have great potential as part of a sustainable, cost-effective agroecological management strategy, which can be integrated with other methods to achieve effective control of FAW.

## Introduction

Fall armyworm (FAW), *Spodoptera frugiperda* (J. E. Smith), is a noctuid polyphagous generalist insect native to tropical and subtropical areas of the Americas. FAW was first reported in India in 2018 when it was observed in a maize crop in the state of Karnataka^1,2^. Since then, FAW has been observed widely across India^3,4^ and other Asian countries^5^.

FAW inflicts significant losses on many agricultural crops^6^. Maize is one of its most preferred hosts, and globally average yield losses of between 17 and 36% resulting from FAW infestation have been observed^7^. FAW infestation results in economic losses of USD 9.4 billion annually across Africa^8^ and increased food insecurity, particularly in low- and middle-income countries where maize is a staple food crop^9^.

The Eastern Gangetic Plain (EGP) spans the Indian states of Bihar, West Bengal, eastern Uttar Pradesh, and Assam as well as parts of the Nepal Terai, and northwestern Bangladesh. The EGP is home to approximately 450 million people, most of whom are resource-poor small and marginal farmers who depend on agriculture for food and livelihood security^10^. The region ranks high in poverty, food insecurity and climatic vulnerability^11–14^. Traditionally, crop production in the EGP is rice-based, with approximately 6.5 m ha each year under either rice-rice or rice–wheat cropping systems, whereby monsoon-season rice is followed by irrigated wheat or rice (boro rice) in the dry season. Only some other crops, such as mustard and pulses, are cultivated after monsoon rice, and jute is also cultivated after boro rice. Over the last two decades, the new cropping system has emerged as rice-maize (covers > 1.0 m ha) in EGP due to its high yield potential in widespread agro-ecologies and climatic conditions of the region. The dry-season maize (winter maize) and spring maize in West Bengal and Bihar increased significantly. Maize is more productive, profitable, and more climatically adaptive and more water, energy, and labor efficient than boro rice^12,14^. Within India, EGP contributes almost 20% of the total maize-grown areas^15^. Thus, FAW is a critical threat to maize farmers within the EGP, and to national food security.

Smallholder farmers generally apply chemical insecticides to control FAW, although many have limited knowledge about appropriate insecticides or how or when to apply them. FAW is resistant to carbamate, organophosphate, and pyrethroid insecticides^16,17^. The use of inappropriate chemicals, methods and/or times of application does not effectively control FAW. However, it also contributes to environmental pollution, negatively affects human health, and increases production costs.

An alternative strategy, integrated pest management (IPM), is a more promising method for smallholder farmers to manage FAW than chemical insecticides^18,19^. IPM encourages the timely and efficient use of both nonchemical and chemical agents to suppress pests within an agroecological environment without completely eliminating them from an agroecosystem, and often by providing conditions in which the pests’ natural enemies thrive^20^. IPM may include specific timing and techniques for crop establishment, nutrient management, variety selection, chemical and nonchemical pesticides, and pest behavior manipulation^21–24^.

There are large numbers of potential FAW parasitoids in the world, and this paves the way to regulate the pest sustainably and economically in farmers’ fields^25,26^. Previous research in the American continents and the Caribbean resulted in the identification of more than 150 parasitoid species that had the potential to suppress FAW in the maize fields and diverse crops habitats, and thus to be a key part of IPM of FAW to minimize crop yield loss^27,28^.

Effective pest suppression through IPM techniques, such as using native parasitoids, is an emerging and potentially vast area of investigation in India and across southern Asia. There has been limited research on FAW parasitoids in southern India^2,29,30^; however no systematic investigation has been conducted in the large maize-growing areas of the Indian EGP into the potential to suppress FAW through IPM, and there is currently very little information for farmers and others on the use of FAW parasitoids to suppress and manage the pest in the maize-producing regions of the EGP.

The aims of this research were: (1) to assess the incidence and severity of FAW in key maize-growing regions within the Indian EGP; (2) to describe the suppression of FAW by endemic pest-parasitoids; and thus (3) to improve the understanding of existing endemic FAW natural enemy species in the Indian EGP, particularly those of the parasitoid complex. To address these aims farmers’ fields were surveyed to quantify the presence of FAW in the EGP and the effect on maize plants. Parasitoids culled from FAW samples were analyzed molecularly and morphologically. This research was conducted in Bihar and West Bengal, India, and has applications elsewhere across the EGP and southern Asia more broadly, in maize-growing areas that are vulnerable to FAW and where parasitoids may form a key part of IPM strategies.

## Results

### FAW distribution, incidence, and severity of damage in maize

FAW was observed in all ten districts surveyed and in maize in three different phenological growth stages. Rice was the preceding crop in nine districts, while farmers of one district, Katihar, grew maize after jute. A significant (*p* < 0.0001) incidence and severity of FAW damage was observed in maize at the early whorl, late whorl, and reproductive stages (Table 1). There were no differences in FAW incidence or severity between the districts, indicating the widespread nature of infestation throughout the EGP. Moreover, interactions between districts and crop growth stages were also not significant in terms of both the incidence and severity of FAW damage to maize crops.

**Table 1:** One-way analyses of variance in FAW incidence and severity of damage to maize crops in ten districts of the Indian Eastern Gangetic Plains.

Y	Source	DF	SS	MSS	F Value	*p* Value
Damage incidence (Plant damage %)	District	9	1644.77	182.75	1.80	0.078
	Stage	2	15,017.88	7508.94	73.34	< 0.0001
	District*stage	18	969.46	53.86	0.53	0.937
Damage severity	District	9	12.57	1.40	1.05	0.408
	Stage	2	128.95	64.47	55.07	< 0.0001
	District*Stage	18	10.55	0.59	0.44	0.975

**Figure 1:**
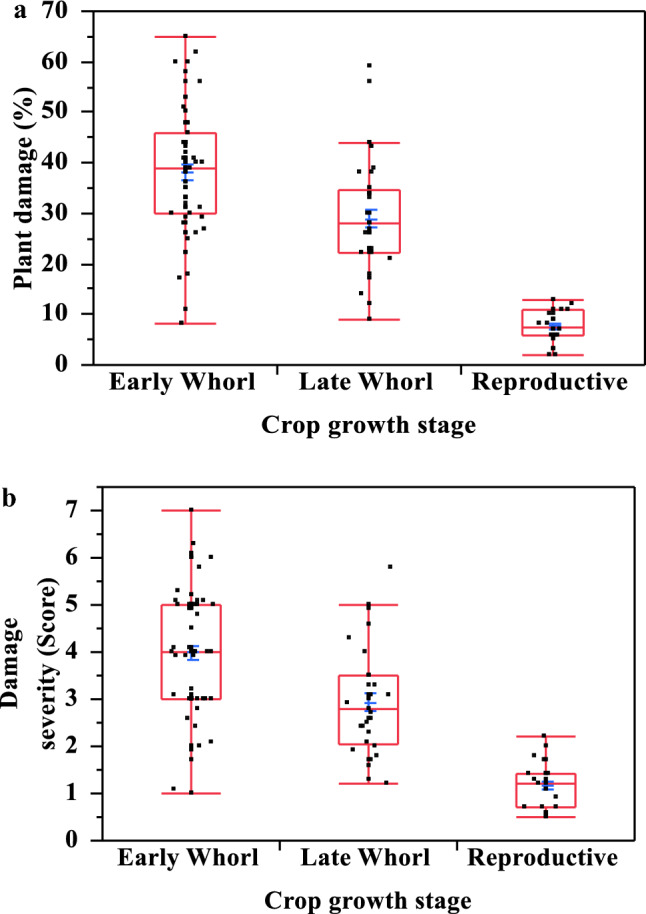
Maize damage (**a**) and its severity score (**b**) by FAW at different crop phenological growth stages (n = 124). Dispersion of observed values of damage incidence (plant damage %) and its severity score in different crop growth stages represented in box plot diagrams. Significant variation (*p* < 0.0001) observed in damage incidence and severity score among crop growth phenological stages viz. early-whorl, late-whorl and reproductive stages. Tukey HSD at 5% interval also confirms significant variation between early-whorl and late-whorl stages (*p* = 0.0002, *p* < 0.0001), early-whorl and reproductive stages (*p* < 0.0001), and late-whorl and reproductive stages (*p* < 0.0001). Early growth stages suffered the highest damage incidence 38.19 ± 1.30% (8–65%), along with a severity score 3.98 ± 0.14 (1.00–7.00).

The highest average FAW damage incidence (38.19%) and average FAW damage severity score (3.98) were observed in maize plants in the early whorl stage, and the greatest plant damage (50–65%) was observed at this time, approximately four to five weeks after sowing (Fig. 1a,b and Supplementary Tables S1). Maize plants at the late whorl stage had moderate damage incidence from FAW (28.97%), with a damage severity score of 2.92. Plants in the reproductive stage had the lowest damage incidence (7.63%) and damage severity score (1.17).

The incidence and severity of FAW infestation were spatially variable across the sample locations within the EGP (Supplementary Table S2). The incidence of average FAW damage varied between 22.71 and 41.29%, with the highest and lowest incidences observed in the Dakshin Dinajpur and Murshidabad districts of West Bengal, where damage severity scores of 3.53 and 3.03, respectively, were recorded. The highest damage severity score (4.02) was recorded in Katihar, Bihar, and the lowest severity score (2.85) was recorded in Coochbehar, West Bengal. Between 18 and 71% of farmers at any sample location applied insecticides. There was no significant difference in the FAW damage incidence or damage severity score in maize in the early whorl stage between crops with or without insecticide application: fields where no insecticide was applied had 38.88% damage incidence and a damage severity score of 4.07, while those where insecticide had been applied had a damage incidence of 37.50% and a damage severity score of 3.89 (Supplementary Tables S3, S4).

**Figure 2:**
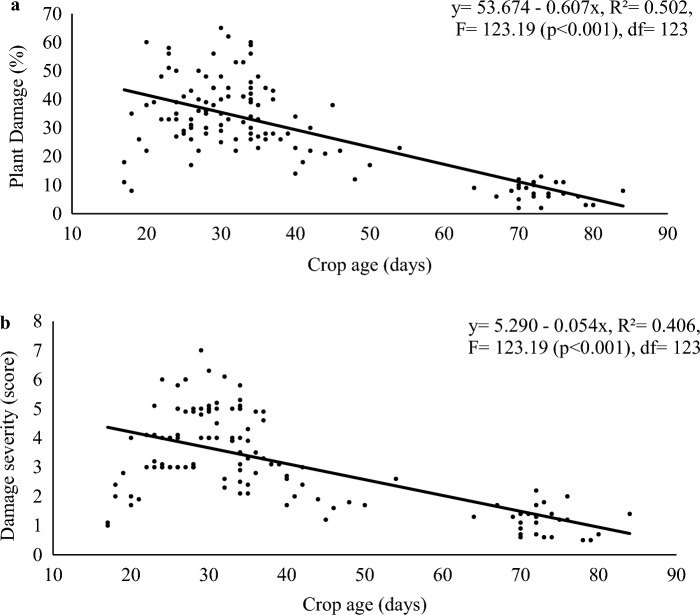
Maize plant damage (**a**) and its severity score (**b**) from FAW at different crop ages (n = 124). Regression analyses indicated a decline in damage incidence (plant damage %) and severity score with increased crop age.

There was a negative correlation between the age of the maize crop and its damage incidence (r =  − 0.71, n = 124 and *p* < 0.0001). A similar trend was observed between maize crop age and damage severity (r =  − 0.64, n = 124 and *p* < 0.0001). Damage incidence and damage severity were positively correlated (r = 0.79, n = 124 and *p* < 0.0001). Regression analyses further confirmed that both damage incidence and severity by FAW declined with the age of the maize plants (Fig. 2a,b).

### Molecular and morphological identification of parasitoids

Overall, 2743 FAW larvae were collected from maize plants across the 93 survey locations in ten districts, and 146 egg masses were collected from 73 locations in nine of these districts. Of the collected larvae, 215 died due to infection by entomopathogens or other unknown factors. The remaining 2528 larvae and the collected egg masses were reared in the laboratory to identify which parasitoid species engaged in parasitism in the Indian EGP. This identification was performed through molecular analysis of the parasitoid species, and comparison with reference accession species in the National Centre for Biotechnology Information, and further morphological identification was performed (Table 2).

**Table 2:** Hymenopteran parasitoids that emerged at different growth stages of FAW collected from maize crops in the Indian EGP and identified through molecular analyses and morphological studies.

Sl. no	Parasitoid name	Host stage affected	GenBank accession No	Similarity with other accessions (%)	Reference accessions
1	*Trichogramma chilonis* (Trichogrammatidae)§	Egg	OQ849581	100	MT219447 OL958558
2	*Telenomus* cf. *remus* (Scelionidae)	Egg	OP288795	99.77	ON923739 MN879316 KY835081 ON737907 OP932000
3	*Chelonus formosanus* (Braconidae)	Egg-Larvae	OP278928	100	MT906644 NC060869 MZ571919
4	*Campoletis chlorideae* (Ichneumonidae)	Larvae	OP269829	98.11	OQ710106 OP898538 MW241326
5	*Charops bicolor* (Ichneunmonidae)	Larvae	OP393900	100	MW506958 OP898533 JF866230
6	*Cotesia ruficrus* (Braconidae)	Larvae	OP288793	99.33	KY837624 KY836674
7	*Microplitis manilae* (Braconidae)	Larvae	OP288794	99.70	HM406523
8	*Microplitis prodeniae* (Braconidae)	Larvae	OR053831	100	MW739950
9	*Euplectrus* spp. (Eulophidae)	Larvae	OR053833	92.04	MT949367
10	*Temelucha* spp. (Ichneumonidae)	Larvae	OQ848503	99.03	MN525186

§within parenthesis represents the family of the species.

The ten parasitoid species identified are hymenopteran: four are from the family Braconidae, three are from the family Ichneumonidae, and one species each was observed from the Trichogrammatidae, Scelionidae, and Eulophidae families. Two parasitoid species were observed infesting FAW egg masses, while eight were observed at the larval stage.

Seven hymenopteran parasitoid species were identified up to the species level, and their morphological keys are presented in Supplementary Table S5. Two parasitoids were found in FAW egg masses: *Telenomus* cf. *remus* Nixon, and *Trichogramma chilonis* Ishii (Supplementary Figs. S1, S2). The parasitoid species identified through molecular analysis as *Telenomus remus* shows 99.77% similarity with the Indian reference accessions (ON923739 and MN879316) and other Asian (KY835081, ON737907 and MT906647) and African accessions (OP93200, MT949366 and MT465126). However, species identification could not be morphologically confirmed, so it is described here as *Te.* cf. *remus*.

Two species of parasitoids belonging to the genus *Microplitis* were observed in FAW larvae: *Microplitis prodeniae* Rao and Kurian (Supplementary Fig. S3) and *Microplitis manilae* Ashmead (Supplementary Fig. S4). Other parasitoid species observed in FAW larvae were two braconids, *Chelonus formosanus* Sonan (Supplementary Fig. S5) and *Cotesia ruficrus* (Haliday) (Supplementary Fig. S6); three ichneumonids, *Campoletis chlorideae* Uchida (Supplementary Fig. S7), *Charops bicolor* (Szepligeti) (Supplementary Fig. S8) and *Temelucha* spp. (Supplementary Fig. S9); and one eulophid; *Euplectrus* spp. (Supplementary Fig. S10a,b). The hatched eggs of *Euplectrus* spp*.* were observed to develop into yellowish-green parasitoid larvae, which attached to the dorsum of the host FAW caterpillar (Supplementary Fig. S10c). The *Euplectrus* spp. larvae had crawled to the underside of the dead FAW host larva and spun a loose cocoon before pupation (Supplementary Fig. S10d).

### Rate and relative abundance of egg and larval parasitoids

Altogether, 2743 FAW larvae were collected from the field. Out of these collected larvae, 215 larvae died because of other factors such as infection by the entomopathogens, parasitic nematodes, physical injuries and other unknown factors. Of the remaining 2528 FAW larvae reared in the laboratory, 234 died by parasitism from one of the eight identified species of larval parasitoids, and of the 146 egg masses collected, two egg parasitoid species parasitized 23 egg masses. Thus, the overall parasitism rates were 9.26% and 15.75% for the FAW larval and egg stages, respectively. Other factors contributed to 8.57% of FAW mortality, as noted at the start of this paragraph. Therefore, the dominance of the hymenopteran group of parasitoids over other factors inflicting mortality at the field level population of the pest was clearly visible.

Of the two egg parasitoids, a greater overall mean parasitism rate (8.90%) of egg masses was observed in *Te.* cf. *remus* and a lower rate (1.37%) in *Tr. chilonis*, while the two parasitoids together had a parasitism rate of 5.48% (Table 3). Egg masses covered with fewer tufts of hair were parasitized by *Tr. chilonis* alone or together with *Te.* cf. *remus*. FAW egg masses collected from four locations, Dakshin Dinajpur, Uttar Dinajpur and Coochbehar in West Bengal and Purnea in Bihar, were not infested by either of these two parasitoid species. The Murshidabad district (West Bengal) had the highest rate of FAW egg mass parasitism (27.27%) by *Te.* cf. *remus*, while both parasitoids together imposed high rates of parasitism on egg masses in the Birbhum (West Bengal, 15.38%) and Kishanganj (Bihar, 25.00%) districts.

**Table 3:** Egg parasitoids and their parasitism rate of FAW egg masses observed across the maize-producing region of the Indian Eastern Gangetic Plains.

Locations	No. of egg masses collected	No. of egg masses parasitized	Observed egg mass parasitoid(s)	Parasitism (%)
Birbhum, West Bengal	26	3	*Te.* cf. *remus*	11.54
		1	*Tr. chilonis*	3.85
		4	*Te.* cf. *remus* + *Tr. chilonis*	15.38
Murshidabad, West Bengal	11	3	*Te.* cf. *remus*	27.27
		1	*Te.* cf. *remus* + *Tr. chilonis*	9.09
Malda, West Bengal	28	5	*Te.* cf. *remus*	17.86
Katihar, Bihar	15	1	*Tr. chilonis*	6.67
Kishanganj, Bihar	12	2	*Te.* cf. *remus*	16.67
		3	*Te.* cf. *remus* + *Tr. chilonis*	25.00
Others (4 districts)	64	0	-	0.00
Overall mean	146	13	*Te.* cf. *remus*	8.90
		2	*Tr. chilonis*	1.37
		8	*Te.* cf. *remus* + *Tr. chilonis*	5.48

*Te.* cf. *remus* = *Telenomus* cf. *remus, Tr. chilonis* = *Trichogramma chilonis.*

**Figure 3:**
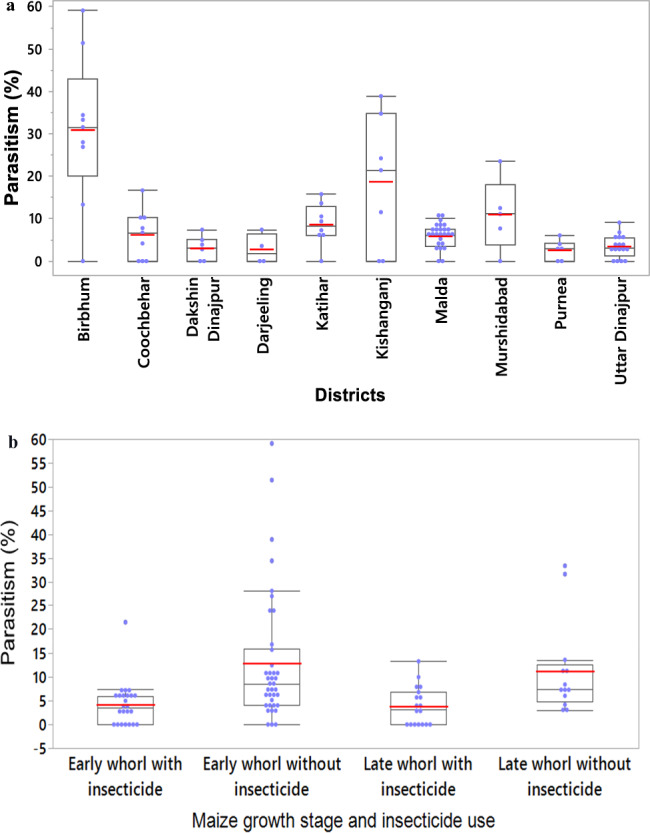
Parasitism rate of FAW (**a**) across the Indian Eastern Gangetic Plains (n = 93, *p* < 0.0001) and (**b**) affected by applying chemical insecticides (n = 93, *p* = 0.0017). Birbhum district showed the highest parasitism rate 30.90 ± 2.55% (0.0–59.09%). Crop growth stages without applying chemical insecticides also recorded significantly higher parasitism rates, with the highest rate of 12.90 ± 1.63% (0.0–59.09%) observed in the early whorl stage.

Across the maize-producing districts in which the study was conducted, there was significant geographic variability (*p* < 0.0001) in the presence of FAW larval hymenopteran parasitoids (Fig. 3a and Supplementary Table S6), and the effect of applying chemical insecticides on the parasitoids (*p* = 0.0017) was observed (Fig. 3b and Supplementary Table S7). The highest average parasitism rate was observed in Birbhum, West Bengal (30.90%), which was significantly higher than that in all other districts except Kishanganj, Bihar (18.70%). Moderate levels of parasitism were observed in Murshidabad (10.97%) and Katihar (8.60%), while all other districts recorded low rates of parasitism, with the lowest (2.56%) observed in Purnea, Bihar.

The application of chemical insecticides was noticeably low in Birbhum, where chemical insecticides had not been applied in approximately 82% of surveyed locations. In contrast, farmers in Dakshin Dinajpur, Uttar Dinajpur and Malda districts relied more on chemicals to control FAW, with between 60 and 70% of the surveyed locations in these districts having applied chemical insecticides.

FAW larvae sampled from early- and late-whorl stage maize without any insecticide applied had the highest parasitism rates (12.90 and 11.28%, respectively). Where insecticides had been applied, parasitism rates were considerably lower: 4.20 and 3.89% in early- and late-whorl stage maize, respectively.

The parasitism rate of individual species varied widely between locations (Table 4). The Birbhum district recorded the highest rate of parasitism by *M. manilae* (13.89%), and additional parasitism from an additional three species (*Ch. formosanus*, 7.54%; *C. chlorideae,* 4.37%; and *Euplectrus* spp., 3.57%). Rates of parasitism by *Cha. bicolor* (7.65%) and *M. prodeniae* (1.76%) were highest in the Kishanganj district, while the parasitoid *Co. ruficrus was* recorded solely in the Malda district, and *Temelucha* spp. only in Murshidabad district. More than one species of parasitoid was observed infesting FAW larvae in all districts except Darjeeling, where *M. manilae* was the only parasitoid found infesting the larval stage of the FAW host.

**Table 4:** Parasitism rates of parasitoid species on FAW larvae collected from different maize-growing regions within the Indian Eastern Gangetic Plains.

Location	Number of larvae reared	Parasitoid observed	Parasitoid family	Parasitism rate (%)
Birbhum, West Bengal	252	*Ch. formosanus*	Braconidae	7.54
		*C. chlorideae*	Ichneumonidae	4.37
		*Euplectrus* spp.	Eulophidae	3.57
		*Cha. bicolor*	Ichneumonidae	4.37
		*M. manilae*	Braconidae	13.89
Murshidabad, West Bengal	97	*C. chlorideae*	Ichneumonidae	3.09
		*Cha. bicolor*	Ichneumonidae	5.15
		*Temelucha* spp.	Ichneumonidae	2.06
Malda, West Bengal	663	*Ch. formosanus*	Braconidae	0.60
		*C. chlorideae*	Ichneumonidae	0.30
		*Co. ruficrus*	Braconidae	0.60
		*Euplectrus* spp.	Eulophidae	0.60
		*Cha. bicolor*	Ichneumonidae	1.06
		*M. prodeniae*	Braconidae	1.21
		*M. manilae*	Braconidae	1.66
Dakshin Dinajpur, West Bengal	166	*Ch. formosanus*	Braconidae	0.60
		*M. manilae*	Braconidae	2.41
Uttar Dinajpur, West Bengal	509	*C. chlorideae*	Ichneumonidae	0.20
		*Euplectrus* spp.	Eulophidae	0.79
		*Cha. bicolor*	Ichneumonidae	0.98
		*M. prodeniae*	Braconidae	0.59
		*M. manilae*	Braconidae	1.18
Darjeeling, West Bengal	98	*M. manilae*	Braconidae	3.06
Coochbehar, West Bengal	150	*C. chlorideae*	Ichneumonidae	2.00
		*M. manilae*	Braconidae	4.00
Katihar, Bihar	231	*Ch. formosanus*	Braconidae	0.87
		*Euplectrus* spp.	Eulophidae	3.03
		*M. prodeniae*	Braconidae	0.87
		*M. manilae*	Braconidae	3.46
Purnea, Bihar	192	*Cha. bicolor*	Ichneumonidae	1.04
		*M. manilae*	Braconidae	1.56
Kishanganj, Bihar	170	*Euplectrus* spp.	Eulophidae	3.53
		*Cha. bicolor*	Ichneumonidae	7.65
		*M. prodeniae*	Braconidae	1.76
		*M. manilae*	Braconidae	10.0

*Ch. formosonus* = *Chelonus formosanus*, *C. chlorideae* = *Campoletis chlorideae*, *Co. ruficrus* = *Cotesia ruficrus*, *Cha. bicolor* = *Charops bicolor*, *M. manilae* = *Microplitis manilae*, *M. prodeniae* = *Microplitis prodeniae.*

**Figure 4:**
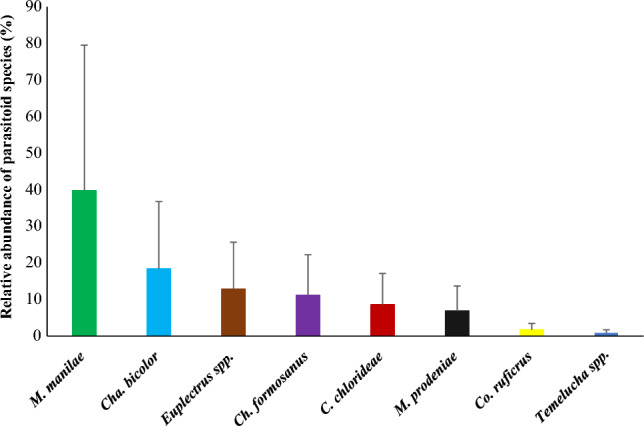
Relative abundance of parasitoids of FAW larvae observed in maize crop in the Indian Eastern Gangetic Plains. Out of 234 parasitized larvae, 93, 43, 30, 26, 20, 16, 4 and 2 larvae died for infestation of *M. manilae*, *Cha. bicolor*, *Euplectrus* spp*.*, *Ch. formosanus*, *C. chlorideae*, *M. prodeniae*, *Co. ruficrus* and *Temelucha* spp*.* respectively. *Microplitis manilae* was recorded as the most dominant larval parasitoid of FAW in the region.

*Microplitis manilae* was the most abundant parasitoid species observed infesting FAW in maize crops across the Indian Eastern Gangetic Plains: it was observed in nine of the ten surveyed districts and had a relative abundance of 39.74% (Fig. 4). Another widely observed species was *Cha. bicolor* (observed in six districts and with a relative abundance of 18.38%); *Euplectrus* spp. (five locations, relative abundance 12.82%); *Ch. formosanus* (four locations, relative abundance 11.11%) and *C. chlorideae* (five locations, relative abundance 8.55%). *Microplits prodeniae*, with a relative abundance of 6.84%, was observed only in a small region in the neighboring districts of Malda and Uttar Dinajpur in West Bengal and Katihar and Kishanganj in Bihar.

## Discussion

In this research we observed greater FAW damage to maize in early growth stages (the early-whorl, V3–V6), with both damage incidence and severity declining in maize plants in the late-whorl and reproductive stages. Similar results have been observed elsewhere in southern Asia^29,31–33^. However, little damage to reproductive tissues may have a greater impact on yield than the foliar damage in the early-whorl growth stages. The crop can compensate for the damage at early stages^34^.

Moderate to high FAW damage was observed in all locations. Two locations, where relatively high use of insecticides was recorded, had greater FAW damage in maize than was observed in locations where no insecticides were applied. The apparent ineffectiveness of the insecticides on FAW suppression may be due to their improper selection, timing, and/or method of application, or to their poor efficiency against FAW. Other research has also demonstrated that the efficacy of chemicals in controlling FAW in maize primarily depends on appropriate insecticide selection and application. Baudron et al.^23^ also reported higher FAW infestation when insecticides were applied than in control plots.

Across ten study locations and from maize crops at different phenological growth stages we observed ten hymenopteran parasitoids: two that attacked FAW egg masses and eight that parasitized FAW larvae. While the mean parasitism rates were highest (12.90%) in FAW collected from early-whorl stage maize grown without insecticide application, the overall mean parasitism rates varied between 2.56 and 30.90% across the collection sites. Elsewhere, parasitism rates in FAW have been observed, between 3.6 and 9.2% in Uganda, and between 13.8 and 39.4% across the Americas^35–38^. The relatively high rate of parasitism observed in this study at Birbhum (30.90%) may have been a result of reduced use of broad-spectrum insecticides relative to other study locations.

We observed that both the parasitoids *Te.* cf. *remus* and *Tr. chilonis* parasitize FAW egg masses: *Te.* cf. *remus* attacks those egg masses that are covered in dense tufts of hair, while *Tr. Chilonis*, which are less hairy: the complimentary effect of these species has also been observed elsewhere^39,40^. *Telenomus remus* has been observed as a main FAW egg parasitoid in America and across Africa in both field and laboratory conditions^25,41^. Other researchers^2,42^ have also observed *Te. remus* parasitizing FAW at field level in India. Another FAW egg parasitoid, *Tr. chilonis*., has been observed elsewhere in maize crops with relatively high parasitism rates in India with a parasitism rate of 12.84–56.25%^29,43^, and in China with parasitism rates of 10.7–31.4%^44^: this parasitoid may also be useful in maize growing regions in eastern India.

Two konobiont endoparasitoids from the genus *Microplitis* were observed in the FAW larvae samples from the survey locations. *Microplitis manilae*, an important parasitoid of the armyworm group^45^, was the most abundant (39.74%) FAW larval parasitoid observed in the Indian EGP, with the highest parasitism rates, of up to 13.89% at Birbhum. A second parasitoid, *M. prodeniae*, was also observed in FAW larvae at four different study locations in the Indian Eastern Gangetic Plains, with parasitism rates between 0.59 and 1.76%. *Microplitis prodeniae* has elsewhere been recognized as a monophagous solitary parasitoid of *Spodoptera litura*^46,47^. The present report revealed that it also effectively parasitized *Spodoptera exigua*^48^. Our observation of *M. prodeniae* parasitizing FAW larvae in the Indian Eastern Gangetic Plains is similar to the findings of researchers from Rajasthan in western India (GenBank accession no. OP898529) and in China (GenBank accession numbers MW739950 and MW250775). As FAW becomes widespread across maize crops in Asia and outcompetes the native Asian armyworm *S. litura* in maize habitats^49^, there is potential for *M. prodeniae*, which has been effective as a biological control of *S. litura*, to also parasitize and thus suppress FAW throughout the region. Further research is needed to confirm this potential.

Other major FAW parasitoids observed in the study region were *Cha. bicolor, C. chlorideae, Ch. formosanus, Temelucha* spp., and *Euplectrus* spp*.,* with relative abundances between 8.55 and 18.38%. *Campoletis chlorideae*, a solitary larval endoparasitoids that is a common parasitoid of FAW in the Indian subcontinent has elsewhere been demonstrated as an effective biological control of FAW^2,29^. *Chelonus formosanus* are egg-larval solitary koinobiont endoparasitoids that have been observed as one of the dominant FAW parasitoids in northern and northwestern India, with a maximum suppression rate of 16.40%^43,50,51^. *Cotesia ruficrus* (Haliday) has been observed to demonstrate considerable parasitism on FAW larvae elsewhere in India^52^, and different parasitoid species of the genus *Cotesia* have also been recorded to attack FAW larvae in other countries^36,53^. *Charops bicolor*, which is an important parasitoid of other obnoxious crop pests, such as yellow stem borer^54^ and *S. litura*^55^, is found to suppress FAW in the eastern states of India. Other parasitoid species of the genus *Charops* were observed as potential parasitoids of FAW by many researchers^35,41^. *Temelucha* spp. are endolarval hymenopteran parasitoids that have previously been shown to be effective against FAW larvae in India^50,56^ and other countries^27^. Similarly, *Euplectrus* spp., a konobiont ectoparasitoid, has been shown to effectively parasitize FAW larvae in India^56^. Other species of the genus *Euplectrus*, such as *E. laphygmae* and *E. platyhypenae*, have also been reported as FAW parasitoids from Africa^57^ and Argentina^37^, respectively.

FAW has particularly large negative impacts on maize growers’ production and profitability across Africa and Asia^8,58^. The extent of damage by FAW is greater outside the native areas due to the absence of naturally occurring biological control agents or native biological control agents of recently invaded areas that have not yet adopted the pest as their host^59^. The search for, and conservation of, parasitoids in agroecologies where FAW has relatively recently arrived is of prime importance to identify cost-effective, ecologically sustainable strategies with which FAW can be controlled in farmers’ fields. Parasitoids may be used effectively as a biological control of an invasive pest through either conservation or augmentative release^30^. Farmers' management practices in maize production are a key component of the cultivation of beneficial parasitoids and their rates of parasitism of FAW. It is likely that insect parasitism of FAW will increase in maize-producing regions of South Asia, as the spread of FAW itself continues. The use of chemical synthetic insecticides has been observed to negatively affect parasitism rates on FAW, and indiscriminate use of chemical insecticides imposes a negative impact on these natural, biological pest control methods^60^. It is imperative that effective, endemic FAW parasitoid species are identified and conserved^38,61^ by using selective insecticides and other agroecological approaches in crop management practices^21,62^.

We identified ten parasitoid species distributed across our sample sites in West Bengal and Bihar: two egg masses and eight larval parasitoids. Of the FAW larval parasitoids, *Microplitis manilae* was the most abundant, while *Te.* cf. *remus* was the most prevalent of the two FAW egg parasitoids. In field conditions where no chemical insecticides were applied, samples taken from maize in the early-whorl growth stage had higher rates of FAW larval parasitism than samples taken from maize in later growth stages. Similarly, FAW egg masses collected from locations where chemical insecticides were not applied had higher rates of parasitism. In contrast, maize plants observed in the early whorl stage to which synthetic chemical insecticides had been applied had comparable FAW damage incidence to that observed in maize plants where no insecticides had been applied.

*Telenomus remus* inflicted natural control of the FAW egg masses in Africa and Asia^25,29,40,41^ and has been found very effective for augmentative biological control in Latin America^63^. It may also be used for augmentative biological control in the newly invaded geographical regions including the study area. *Trichogramma chilonis* is already in use in India for augmentative release of other lepidopteran pests in paddy and sugarcane fields^64,65^ and may also be tested against FAW in farmers’ fields. Further attempts to identify and select suitable natural enemies of FAW for enhanced biological control in maize should consider two main characteristics: high efficacy to the host; and high host specificity to avoid nontarget effects^34^. *Microplitis manilae*, which generally infests *Spodoptera* spp. may also be of use and should be further investigated. At the same time, *Microplitis prodeinae*, which was considered as solitary parasitoid of *S. litura*, started adopting FAW as its host. All ten parasitoid species observed in this study naturally control the FAW population in the maize field of eastern India and this parasitoid complex may be the game changer in the near future. The conservation of these parasitoids in the maize habitats to achieve effective and sustainable suppression of FAW at early growth stages of the crop requires a combined application of all possible agro-ecological management strategies, including the use of botanicals, biopesticides, intercrops, trap crops, and so on, without using chemical pesticides in the first 30 to 45 days of the crop growth stages.

## Method

All experimental research on cultivated plants and insects described here complied with relevant institutional, national and international guidelines and legislation.

### Fall armyworm survey and assessment of damage

**Figure 5:**
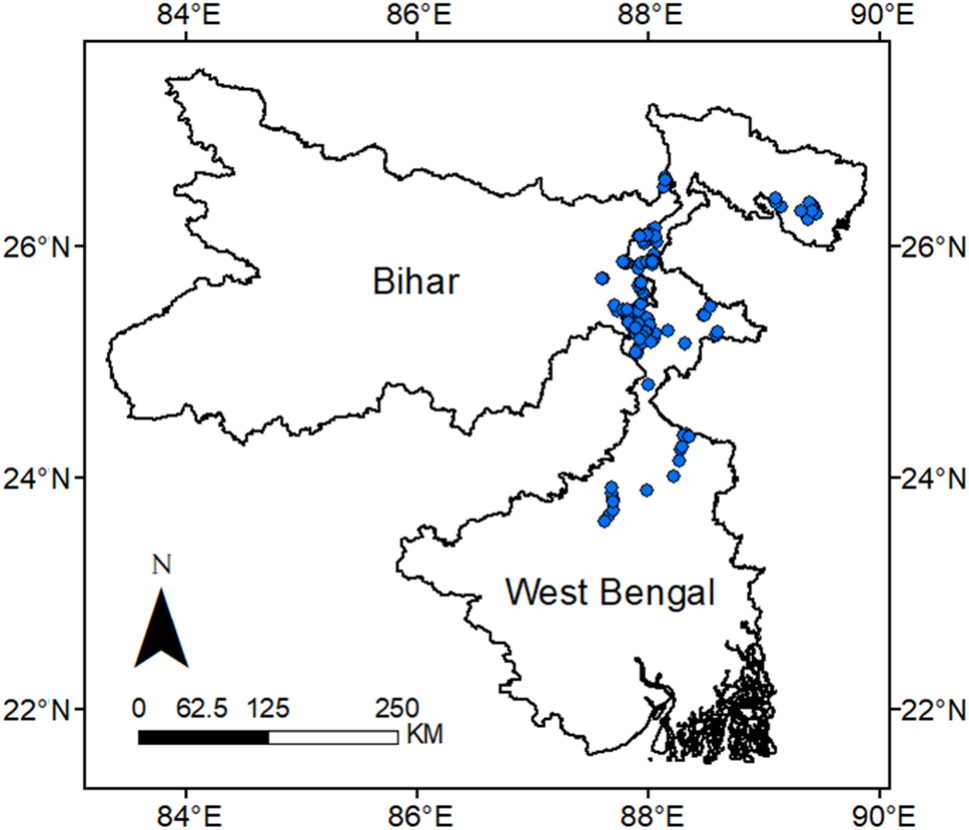
GPS coordinates of survey locations in the eastern gangetic plains of India.

Farmers’ fields were surveyed between October 2021 and March 2022 to quantify the incidence and effects of FAW on farmers’ maize crops in two Indian states, West Bengal and Bihar. The survey was conducted in over 142 different locations in ten districts of these two states, in a north–south gradient at approximately 23.5–26.5°N and centered around 88°E (Fig. 5). The survey covered approximately 40% of the maize cultivated area in Bihar and West Bengal.

FAW infestation was determined through the presence of fresh frass and feeding injuries on maize whorls and leaves. One hundred twenty-four scouting locations had FAW incidence. The methodology of McGrath et al.^66^ was followed, examining maize plants for ‘W Scouting patterns’ in the early- (VE to V6: emergence to six leaves stage) and late- (V7 to VT: seven leaves to initiation of tasseling stage) whorl phenological growth stages, and for ‘ladder patterns’ in the reproductive (tasseling and silking) stages. At each sampling location, five spots of 4.0 m row length with 3 rows were randomly selected from within a maize field. Twenty plants were randomly selected from each spot and used to quantify the maize infestation damage by expressing the number of plants infested by FAW out of the hundred plants examined as a percentage.

Ten maize plants (two each from the five spots) were randomly selected from each sampling location, and the severity of FAW damage on the newly grown plant parts (i.e., whorls and furls) was quantified using the simple day-independent scale described by Davis and Williams^67^ and suggested by Toepfer et al.^68^, which uses Eq. (1):

1 $$\mathrm{I }= [\sum (\mathrm{n x v})/(\mathrm{N x Z})] \times 100$$

where I = severity of FAW damage, Z = highest possible damage score (here 9), N = number of plants observed, n = number of plants that have a ‘v’ value, v = value (score) of the crop damage, where 0 is no visible signs of damage and 9 is almost total destruction of whorl and furled leaves.

FAW egg masses and larvae were collected from early- and late-whorl stages of maize plants at all locations where they were observed and grouped into those that had or had not been sampled from maize treated with chemical insecticides based on information gathered from farmers. FAW gathered from maize where insecticides had been applied at least 25 days prior were deemed to be in the “noninsecticide group”; those gathered from maize where insecticides had been applied within 25 days were the “insecticide group”. Mostly the first to fourth larval instars were collected through semidestructive sampling by pulling apart the damaged leaf whorls. Egg masses were collected from 7- to 21- day-old plants. Farmer’s cropping system management information was also collected from each survey sample plot.

### Collection of FAW and rearing for emergence of parasitoids

The field collected FAW egg masses and larvae were morphologically identified^69^ and reared under laboratory conditions following standard protocols^52^. The larvae were placed individually in 100 ml transparent plastic rearing containers with small pin holes at the top cover to allow ventilation while containing the larvae. Maize leaves were rinsed with double distilled water, cut into small pieces, and transferred into rearing containers. Filter paper (Whatman grade 1, size 110 mm) was placed at the bottom of the containers to absorb excess moisture produced by the maize leaves and larvae, and frass. The larvae were reared on maize leaves in the laboratory under room conditions (27 ± 3 °C, 75 ± 5% RH, and 12:12 h photoperiod). The larvae were examined every 24 h for emerged parasitoids which were collected, placed in 70% ethanol, and then subjected to molecular identification. Parasitoids were photographed using a stereo zoom microscope (make: Lieca S9i).

### Molecular identification of parasitoids

High-quality DNA was obtained from parasitoids and then submitted to the National Centre for Biotechnology Information to determine GenBank accession numbers and match them to reference accessions through BLAST analysis. DNA was extracted from the parasitoid insects using Qiagen D Neasy^®^ kits, following the manufacturer’s protocol. The DNA extracts were subjected to polymerase chain reaction (PCR) amplification of a 700 bp region near the 5′ terminus end of the COX1 gene following the standard protocol^70^. The primers used were as follows: forward primer (LCO 1490: 5′- GGTCAACAAATCATAAAGATATTGG-3′), and reverse primer (HCO 2198: 5′- TAAACTTCAGGGT GACCAAAAAATCA-3′). PCRs were carried out in 96-well plates with a 50 µL reaction volume containing: 5 µL GeNeiTM Taq buffer, 1 µL GeNeiTM 10 mM dNTP mix, 2.5 µL (20 pmol/µL) forward primer, 2.5 µL (20 pmol/µL) reverse primer, 1 µL GeNeiTM Taq DNA polymerase (1 U/µL), 2 µL DNA (50 ng/µL), and 36 µL sterile water. Thermocycling consisted of an initial denaturation at 95 °C for 3 min, followed by 35 cycles of denaturation at 95 °C for 30 s, annealing at 50 °C for 30 and extension at 72 °C for 45 s followed by another 3 min at 72 °C. PCR analysis was performed using a C1000™ Thermal Cycler. The amplified products were analyzed by 1.5% agarose gel electrophoresis. The amplified products were purified to remove contaminants and then sequenced using a BDT v3.1 Cycle sequencing kit on an ABI 3730xl Genetic Analyzer. The consensus sequence of the COI gene was generated from forward and reverse sequence data using aligner software. The COI gene sequence was used to carry out BLAST with the ‘nr’ database of NCBI GenBank database. Using a maximum identity score, the first ten sequences were selected and aligned using a multiple alignment software program. A cluster W Distance matrix was generated using an RDP database, and the phylogenetic tree was constructed using MEGA 6. The amplified products were sequenced by Barcode Biosciences Private Limited, Bengaluru, India. This was further compared with the BOLD (Barcode of Life Data System) to confirm their similarity with the other barcoded parasitoid specimens across the world.

### Morphological study of parasitoids

Parasitoids were identified, and sample specimens were preserved in the laboratories of Visva Bharati University and the National Insect Museum of the Indian Council of Agricultural Research-National Bureau of Agricultural Insect Resources, India. The morphological analysis of two parasitoid species under the genus *Microplitis* was conducted using the taxonomic keys outlined by Gupta^71^, and species identification was determined using previously published descriptions^72,73^. The standard morphological descriptions were also followed for *Co. ruficrus*^74^; *Cha. bicolor*^75^; *C. chlorideae*^76^; *Che. formosanus*^77^; *Te.* cf. *remus*^78^; and *Tr. chilonis*^79,80^. The morphology of the genera *Temelucha* and *Euplectrus* were matched with the general description proposed by Townes^81^ and Hansson et al.^82^, respectively.

### Parasitoid effectiveness

The number of FAW larvae collected was calculated by subtracting from the total number of larvae those that had died from other causes besides parasitoids, such as infection by entomopathogens, predators, insecticides, physical injury, or unknown causes. Next, the parasitism rate (PR) and relative abundance (RA) were calculated using the methodology of Pair et al.^61^ and Agboyi et al.^83^, respectively. The percentage of FAW killed by parasitoids was calculated for each location by dividing the number of parasitoids that emerged by the total number of FAW larvae collected and multiplying by 100. The PR of each parasitoid species was calculated using Eq. (2):

2 $$PR=\frac{Lp}{TL}\times 100$$

where *Lp* is the number of FAW larvae parasitized and *TL* is the total number of FAW larvae collected.

The RA of each species was determined using Eq. (3):

3 $$RA=\frac{Ni}{Nt} \times 100$$

where *Ni* is the number of individuals of a given species and *Nt* is the total number of all parasitoids that were recorded.

### Data analysis

Data were graphed and examined for correlations and regressions using R version 4.3.0. Analysis of variance (ANOVA) was conducted using the statistical analysis system (SAS) software (Version 9.4) on plant damage incidence and damage severity, using field plot location, crop growth stage and their interaction as factors. Similarly, ANOVA was also performed on the parasitism rate across the locations and the impact of chemical insecticides on the parasitism rate at different crop growth stages. The normality assumption of analysis of variance (ANOVA) was tested using the Shapiro Wilk test^84^. Pooled treatment adjusted means under different parameters were compared using Tukey’s honest significant difference (HSD) test (*p* ≤ 0.05).

### Supplementary Information

Supplementary Information.

## Data Availability

The dataset generated through molecular analysis during the current study is available with the following links: 1. *Trichogramma chilonis* (OQ849581): https://www.ncbi.nlm.nih.gov/sites/entrez?cmd=Search&db=nucleotide&term=OQ849581.1&dopt=GenBank. 2. *Telenomus* cf. *remus* (OP288795): https://www.ncbi.nlm.nih.gov/sites/entrez?cmd=Search&db=nucleotide&term=OP288795.1&dopt=GenBank. 3. *Chelonus formosanus* (OP278928): https://www.ncbi.nlm.nih.gov/sites/entrez?cmd=Search&db=nucleotide&term=OP278928.1&dopt=GenBank. 4. *Campoletis chlorideae* (OP269829): https://www.ncbi.nlm.nih.gov/sites/entrez?cmd=Search&db=nucleotide&term=OP269829.1&dopt=GenBank. 5. *Charops bicolor* (OP393900): https://www.ncbi.nlm.nih.gov/sites/entrez?cmd=Search&db=nucleotide&term=OP393900.1&dopt=GenBank. 6. *Cotesia ruficrus* (OP288793): https://www.ncbi.nlm.nih.gov/sites/entrez?cmd=Search&db=nucleotide&term=OP288793.1&dopt=GenBank. 7. *Microplitis manilae* (OP288794): https://www.ncbi.nlm.nih.gov/sites/entrez?cmd=Search&db=nucleotide&term=OP288794.1&dopt=GenBank. 8. *Microplitis prodeniae* (OR053831):.https://www.ncbi.nlm.nih.gov/sites/entrez?cmd=Search&db=nucleotide&term=OR053831.1&dopt=GenBank. 9. *Euplectrus* spp. (OR053833): https://www.ncbi.nlm.nih.gov/sites/entrez?cmd=Search&db=nucleotide&term=OR053833.1&dopt=GenBank. 10. *Temelucha* spp. (OQ848503): https://www.ncbi.nlm.nih.gov/sites/entrez?cmd=Search&db=nucleotide&term=OQ848503.1&dopt=GenBank. Supplementary information is also available with http://nature.com.
