# Supplementary material for: Hymenopteran parasitoid complex and fall armyworm: a case study in eastern India
Source: Sci Rep. 2024 Feb 18;14:4029. doi: 10.1038/s41598-024-54342-z (PMC10874954; doi:10.1038/s41598-024-54342-z)
Supplement: Supplementary file 1 — Supplementary Information. [file 41598_2024_54342_MOESM1_ESM.pdf]

Hymenopteran parasitoid complex and fall armyworm: A case study in eastern India

Subhajit Pal[1], Swarnali Bhattacharya[1*], Tapamay Dhar[2], Ankita Gupta[3], Arunava Ghosh[2], Sandip Debnath[1], Nikhitha Gangavarapu[1,4], Prajna Pati[1,5], Nilanjana Chaudhuri[2], Hirak Chatterjee[1], Sabita Kumar Senapati[2], Prateek Madhab Bhattacharya[2], Mahesh Kumar Gathala[6], Alison M. Laing[6]

1. Visva-Bharati University, Santiniketan, Birbhum, West Bengal - 731235, India
2. Uttar Banga Krishi Viswavidyalaya (UBKV), Pundibari, Coochbehar, West Bengal - 736165, India
3. ICAR-National Bureau of Agricultural Insect Resources (NBAIR), Bengaluru, Karnataka - 560024, India
4. University of Nebraska-Lincoln, Lincoln, NE - 68583-0816, USA
5. Siksha 'O' Anusandhan Deemed to be University, Bhubaneswar, Odisha - 751030, India
6. International Maize and Wheat Improvement Center (CIMMYT), Dhaka - 1212, Bangladesh

*Corresponding Author's email:
drbhattacharyaswarnali@gmail.com

Table S1: Damage incidence and severity of FAW at different phenological growth stages of maize in Indian Eastern Gangetic Plains

Crop Growth Stage	No. of Observations	Damage incidence (Plant damage %)	Damage severity (Score)
Early whorl	67	38.19 ± 1.30^{A}<br>(8.00-65.00)	3.98 ± 0.14^{A}<br>(1.00-7.00)
Late whorl	33	28.97 ± 1.85^{B}<br>(9.00-59.00)	2.92 ± 0.20^{B}<br>(1.20-5.80)
Reproductive	24	7.63 ± 2.16^{C}<br>(2.00-13.00)	1.17 ± 0.23^{C}<br>(0.50-2.20)
df		121	121
F value		73.34	55.07
p value		<0.0001	<0.0001
Exact level of significance for comparison of means between early whorl & late whorl by using Tukey HSD Test		0.0002	<0.0001
Exact level of significance for comparison of means between early whorl & reproductive by using Tukey HSD Test		<0.0001	<0.0001
Exact level of significance for comparison of means between late whorl & reproductive by using Tukey HSD Test		<0.0001	<0.0001

damage incidence and severity expressed as mean ± SE with the range in parentheses; Crop growth stages not connected with the same letter are significantly different

Table S2: Damage incidence and severity of FAW on maize across the survey districts in Indian Eastern Gangetic Plains

District	No. of Observations	Locations with insecticides application (%)	Damage incidence (Plant damage %)	Damage Severity (Score)
Birbhum, West Bengal	11	18.18	34.91 ± 5.77 (10.00-62.00)	3.08 ± 0.38 (1.00-5.00)
Murshidabad, West Bengal	7	28.57	22.71 ± 5.64 (2.00-40.00)	3.03 ± 0.76 (0.60-6.00)
Malda, West Bengal	32	68.75	25.63 ± 1.93 (3.00-48.00)	2.94 ± 0.22 (0.70-5.20)
Dakshin Dinajpur, West Bengal	7	71.43	41.29 ± 6.78 (7.00-65.00)	3.53 ± 0.59 (1.20-6.30)
Uttar Dinajpur, West Bengal	22	59.09	32.64 ± 3.67 (6.00-60.00)	2.99 ± 0.40 (0.70-6.00)
Darjeeling, West Bengal	5	40.00	41.20 ± 8.84 (11.00-60.00)	3.52 ± 0.58 (2.00-5.10)
Coochbehar, West Bengal	13	53.85	27.54 ± 4.45 (3.00-46.00)	2.85 ± 0.44 (0.50-5.30)
Katihar, Bihar	10	40.00	30.90 ± 5.21 (6.00-56.00)	4.02 ± 0.52 (1.30-6.00)
Purnea, Bihar	9	33.33	29.33 ± 4.81 (11.00-53.00)	3.52 ± 0.61 (1.10-6.10)
Kishanganj, Bihar	8	25.00	23.88 ± 4.74 (2.00-40.00)	3.14 ± 0.63 (0.70-5.10)
df			114	114
F value			1.80	1.05
p value			0.08	0.41

Damage incidence and severity expressed as mean ± SE with the range in parentheses

Supplementary Table S3: Impact of insecticide application on FAW damage at different phenological growth stages of maize in Indian Eastern Gangetic Plains

Maize growth stage and insecticide presence	No. of observations	Damage incidence (Plant damage %)	Exact level of significance for mean comparisons of all pairs by using Tukey HSD Test					
			Early whorl with insecticide	Early whorl without insecticide	Late whorl with insecticide	Late whorl without insecticide	Reproductive with insecticide	Reproductive without insecticide
Early whorl with insecticide	27	37.50 ± 2.05^{A} (18.00-65.00)	-	0.988	0.001	0.675	<0.0001	<0.0001
Early whorl without insecticide	40	38.88 ± 1.69^{A} (8.00-62.00)	0.988	-	0.021	0.313	<0.0001	<0.0001
Late whorl with insecticide	20	26.09 ± 2.38^{B} (9.00-44.00)	0.001	0.021	-	0.811	<0.0001	<0.0001
Late whorl without insecticide	13	31.85 ± 2.96^{AB} (22.00-59.00)	0.675	0.313	0.811	-	<0.0001	<0.0001
Reproductive with insecticide	11	6.95 ± 3.21^{C} (2.00-13.00)	<0.0001	<0.0001	<0.0001	<0.0001	-	0.999
Reproductive without insecticide	13	8.31 ± 2.96^{C} (3.00-12.00)	<0.0001	<0.0001	<0.0001	<0.0001	0.999	-
df		118						
F value		29.53						
p value		<0.0001						

Damage incidence (Plant damage%) expressed as mean ± SE with the range in the parentheses; Crop growth stages and insecticide presence not connected by the same letter are significantly different

Supplementary Table S4: Impact of insecticide application on FAW damage severity at different phenological growth stages of maize in Indian Eastern Gangetic Plains

Maize growth stage and insecticide presence	No. of observations	Damage severity (score)	Exact level of significance for mean comparisons of all pairs by using Tukey HSD Test					
			Early whorl with insecticide	Early whorl without insecticide	Late whorl with insecticide	Late whorl without insecticide	Reproductive with insecticide	Reproductive without insecticide
Early whorl with insecticide	27	3.89 ± 0.22^{A} (1.10-6.30)	-	0.978	0.020	0.451	<0.0001	<0.0001
Early whorl without insecticide	40	4.07 ± 0.18^{A} (1.00-7.00)	0.978	-	0.001	0.131	<0.0001	<0.0001
Late whorl with insecticide	20	2.68 ± 0.26^{B} (1.20-5.00)	0.020	0.001	-	0.938	0.001	0.008
Late whorl without insecticide	13	3.16 ± 0.32^{AB} (1.60-5.80)	0.451	0.131	0.938	-	0.0001	0.001
Reproductive with insecticide	11	0.96 ± 0.34^{C} (0.50-1.80)	<0.0001	<0.0001	0.001	0.0001	-	0.967
Reproductive without insecticide	13	1.38 ± 0.32^{C} (0.60-2.20)	<0.0001	<0.0001	0.008	0.001	0.967	-
df		118						
F value		22.62						
p value		<0.0001						

Damage severity (score) expressed as mean ± SE with the range in the parentheses; Crop growth stages and insecticide presence not connected by the same letter are significantly different

Supplementary Table S5: Morphological keys of Hymenopteran parasitoids identified up to species level

Sl. No.	Parasitoid Name	Brief Diagnosis
1	*Trichogramma chilonis* Ishii (Family Trichogrammatidae)	Male with blackish mesoscutum and metasoma. Antennal hairs sharply tapering and moderately long, longest being nearly two and half times the maximum width of flagellum. Fringe on tornus of forewing about one-sixth width of wing. Females are yellow with first three abdominal terga black. Antenna clubbed with few short hairs on flagellum. Ovipositor as long as or slightly longer than hind tibia.
2	*Campoletis chlorideae* Uchida (Family Ichneumonidae)	Female body colour black; metasoma yellowish brown with median black infuscation in second to sixth metasomal tergites dorsally. Fore and mid legs largely yellowish brown; hind coxa concolourous with mesosoma, hind tibia with dark brown pre basal and one third apical region, remainder yellow. Apical margin of clypeus with median tooth; inner eye margins weakly indented. Forewing areola joining second recurrent vein before the middle point. Metasoma with moderately slender petiole, stouter behind. Ovipositor sheaths well exserted and about 0.64× as long as hind tibia.
3	*Charops bicolor* (Szépligeti) (Family Ichneumonidae)	Body black, antennae dark brown to black; yellow orange markings on the antennal bases, legs, and metasoma; legs more or less yellowish; hind femur yellowish brown; base of first tarsus yellow; petiole fully reddish; metasoma yellowish brown. Eye distinctly emarginate. Pronotum narrow with a deep groove. Petiole long and slender, without lateral groove. Fore wing venation without areolet. Ovipositor short, about equal to the apical depth of metasoma.
4	*Chelonus formasanus* Sonan (Family Braconidae)	Body colour black; fore and mid legs yellowish orange except coxa, trochanter and infuscation at base of femur black; hind leg black with apex of hind femur, middle one third of tibia and base of tibia yellowish white; tegula black with yellowish apical tip; wings slightly infuscate, metasomal carapace with a pair of subbasal ivory lateral spots, remainder black.
5	*Cotesia ruficrus* (Haliday) (Family Braconidae)	Body colour black; legs yellowish brown except black hind coxae, tegulae, apices of hind femora and hind tibia. Mesonotum posteriorly coarsely and closely punctate, scutellum strongly punctate, punctures well separated from each other. Mesopleuron mostly smooth and shining posteriorly and above. Forewing with the first abscissa of radial vein almost equal in length to transverse cubital vein. Hind coxae mostly rugulose. Propodeum coarsely rugose. First and second tergites of metasoma rugulose, remaining tergites smooth. Ovipositor sheaths well exerted but not longer than hind tibial spurs.
6	*Microplitis manilae* (Ashmead) (Family Braconidae)	Notauli faintly indicated; T1 (first tergite) parallel sided, slight narrowed at apex with apical swelling; finely rugose punctate in posterior half except apical patch; T1 of metasoma black, laterotergites of T1 and T2 (second tegite0 yellow brown. T2 with small brown black median field indicated by faint oblique grooves. Latero-tergites yellowish brown; T3 (third tergite) with mixture of yellow, brown and black patches; remainder of tergites dark brown to black; hind tibia with median one third pale testaceous to white, apical 1/3rd with black infuscation; median length of T3 more than T2.
7	*Microplitis prodeniae* Rao & Kurian (Family Braconidae)	Hind femur reddish brown (darker dorsally and apically); tibia medially pale testaceous to white, apical one third dark brown; one fourth extreme base with black infuscation. Notauli complete; scutellar sulcus with seven distinct costulae; scutellum dull and rugose; propodeum with median longitudinal carina surrounded by coarse rugosity; transverse carinae present; T1 of metasoma black (except brown apex); anterior laterotergites light brown, first tergite with the sides parallel (narrow at apex), twice as long as broad, broadly excavate up to the basal two-third in the middle, rugose except for shining apex; second and third tergite reddish brown; smooth and shiny; T2 dark brown at median field, median fields without slanting margins; T3 with a mix of red, brown and black colour, black at lateral sides; rest of the metasoma smooth, shiny and black.

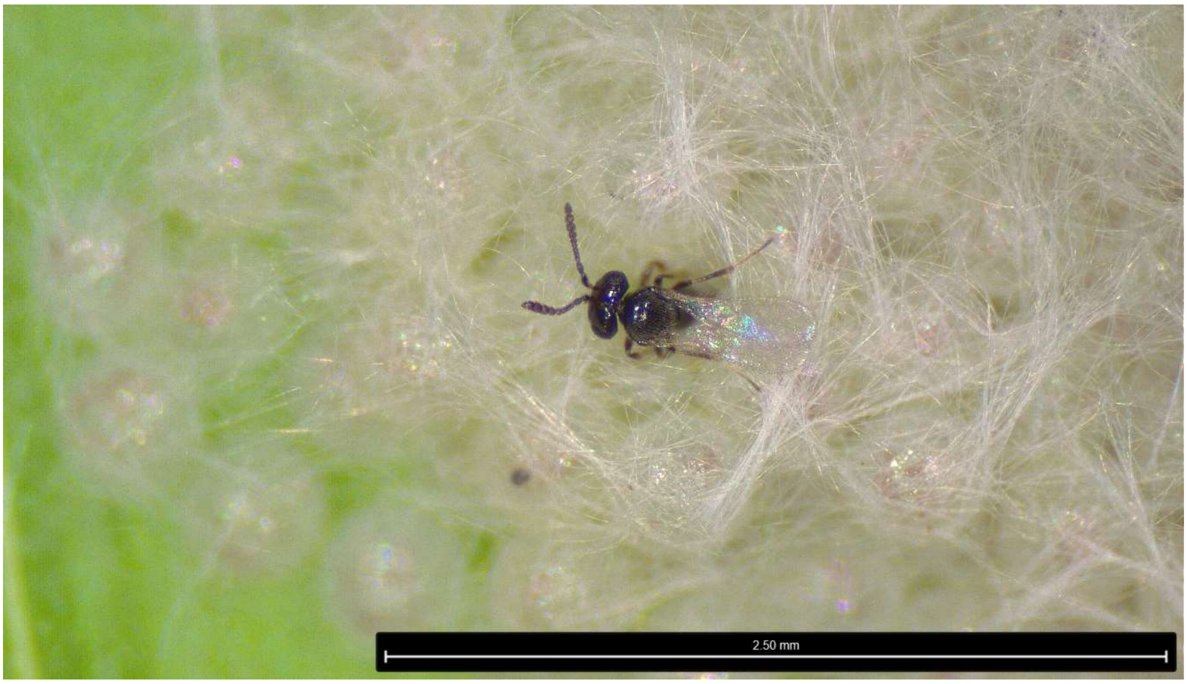

Supplementary Figure S1: Dorsal view of *Telenomus* cf. *remus* Nixon

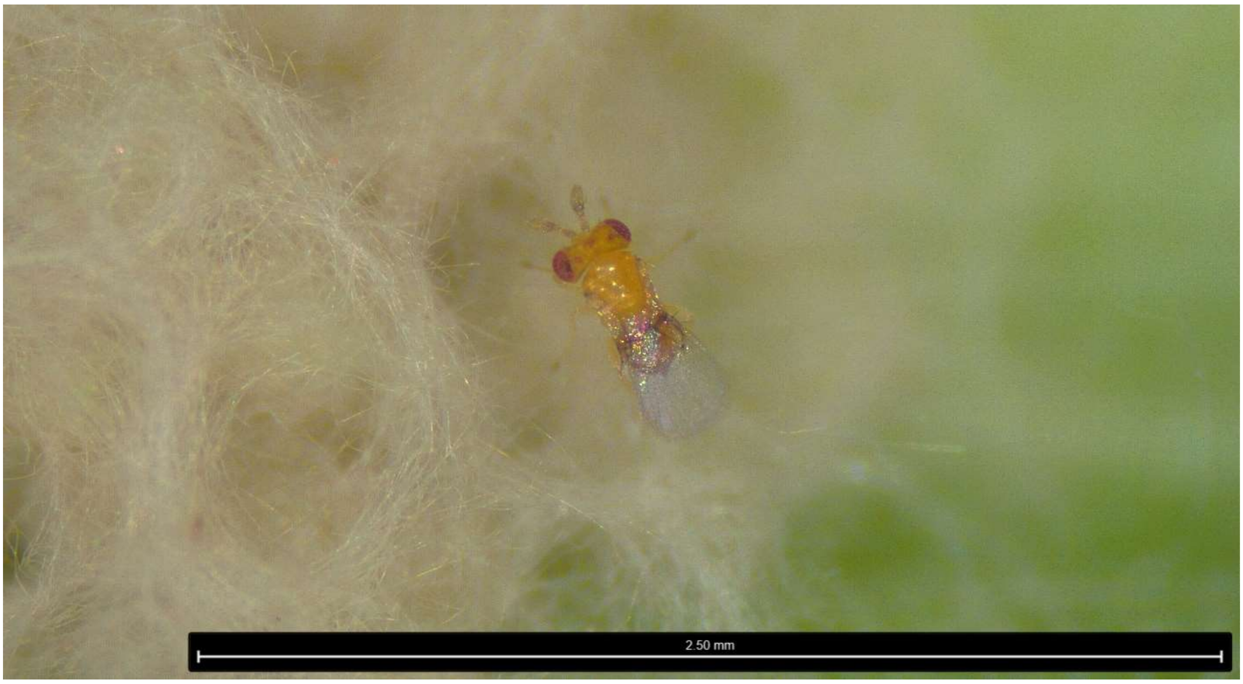

Supplementary Figure S2: Dorsal view of *Trichogramma chilonis* Ishii

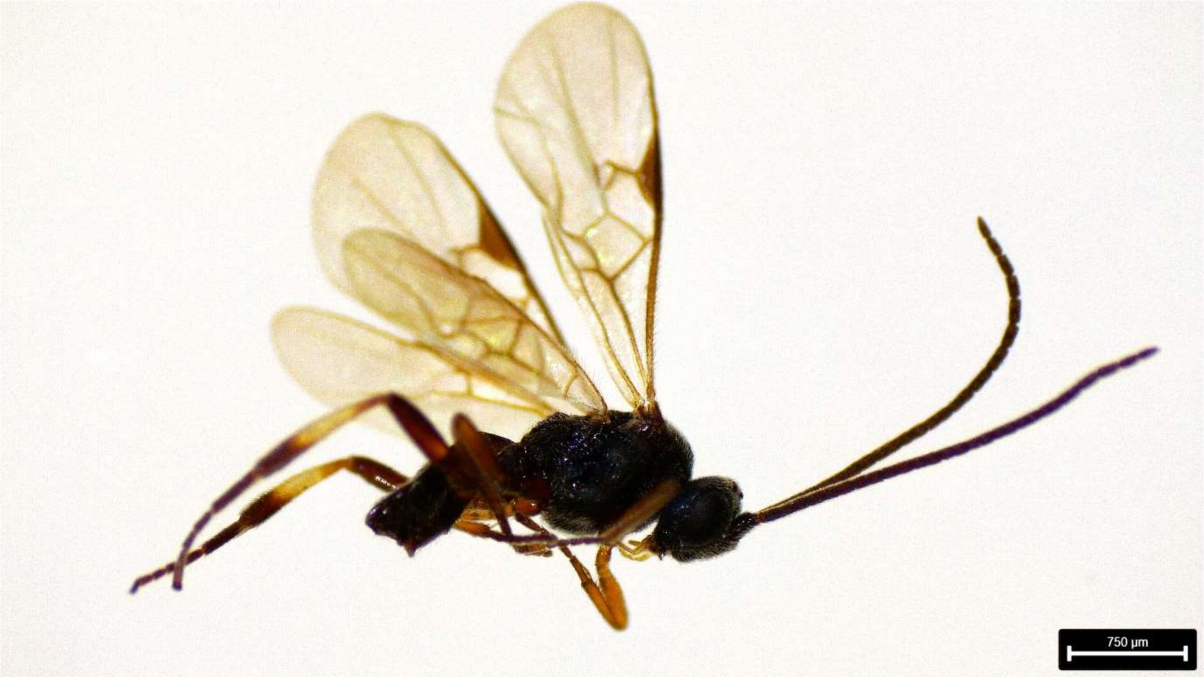

Supplementary Figure S3: Lateral view of *M. prodeniae* Rao and Kurian

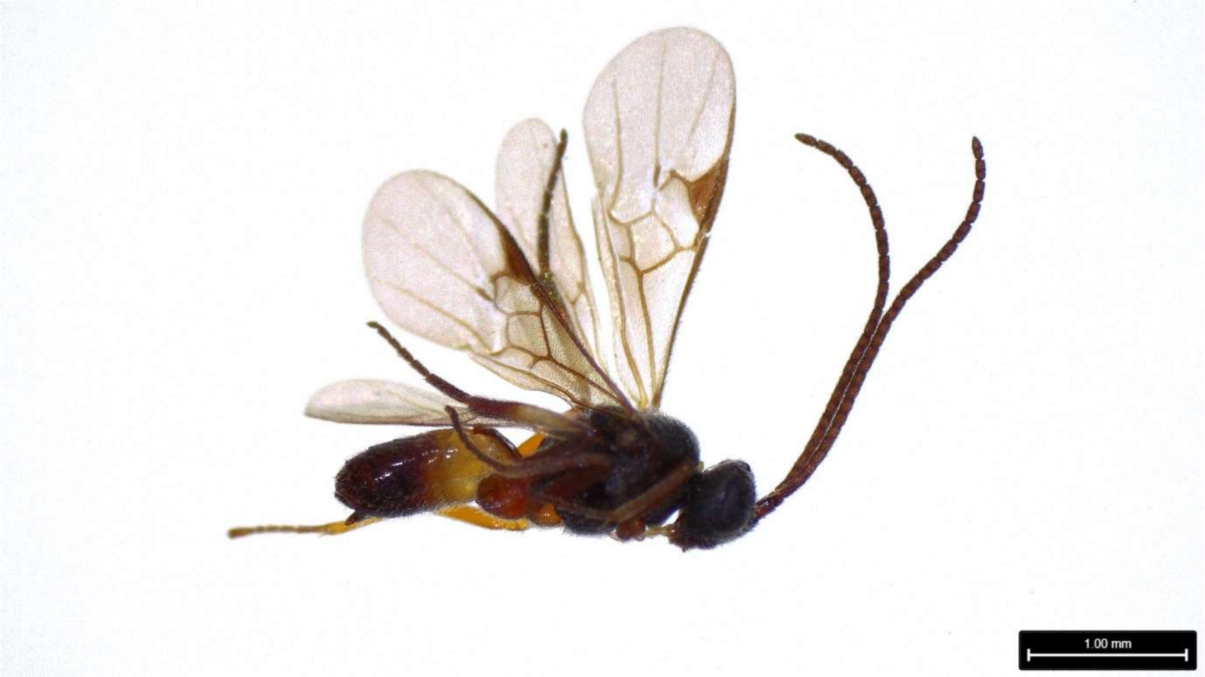

Supplementary Figure S4: Lateral view of *M. manilae* Ashmead

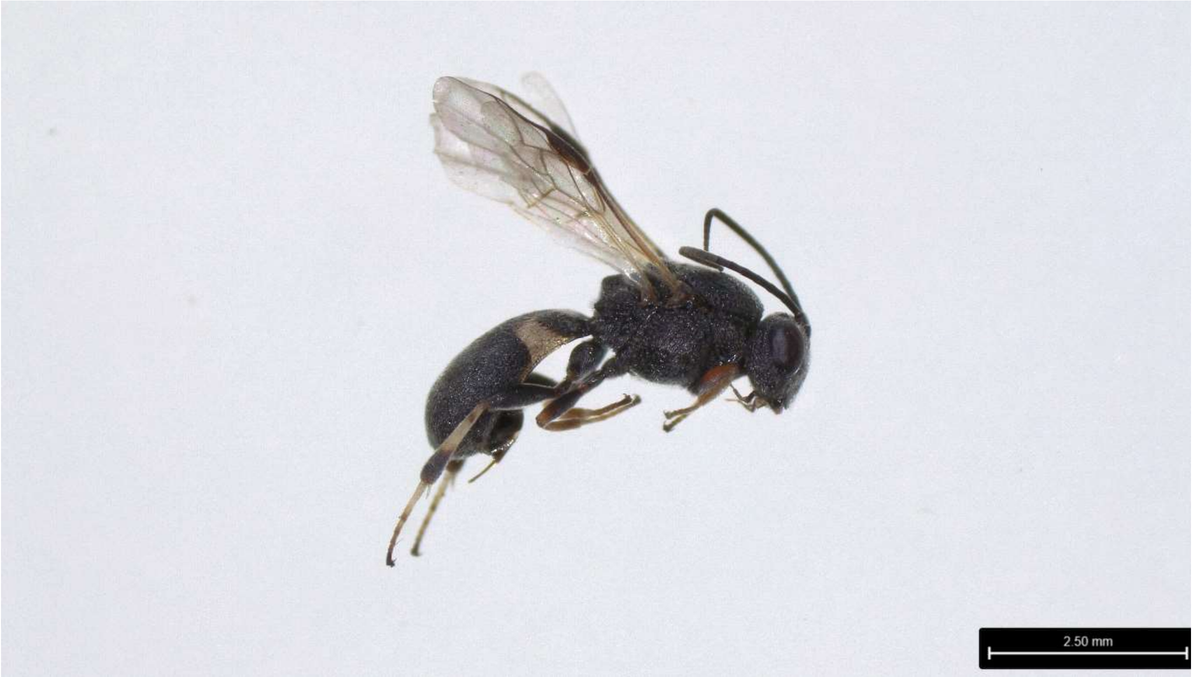

Supplementary Figure S5: Lateral view of *Chelonus formosanus* Sonan

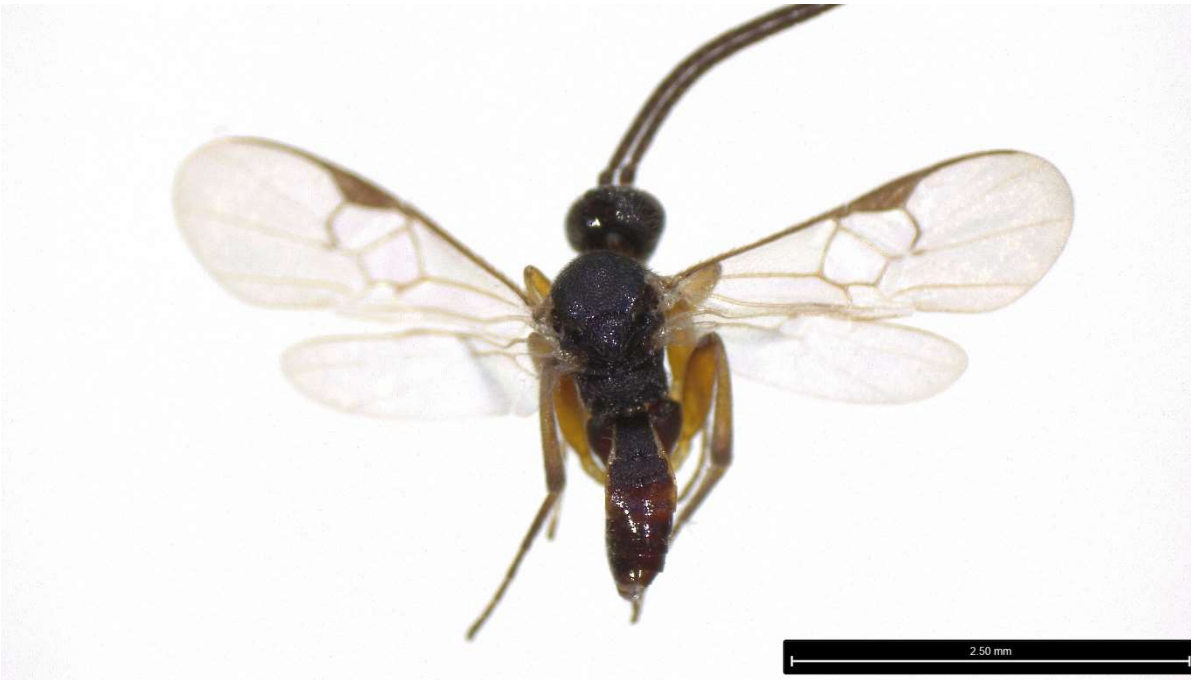

Supplementary Figure S6: Dorsal view of *Cotesia ruficrus* (Haliday)

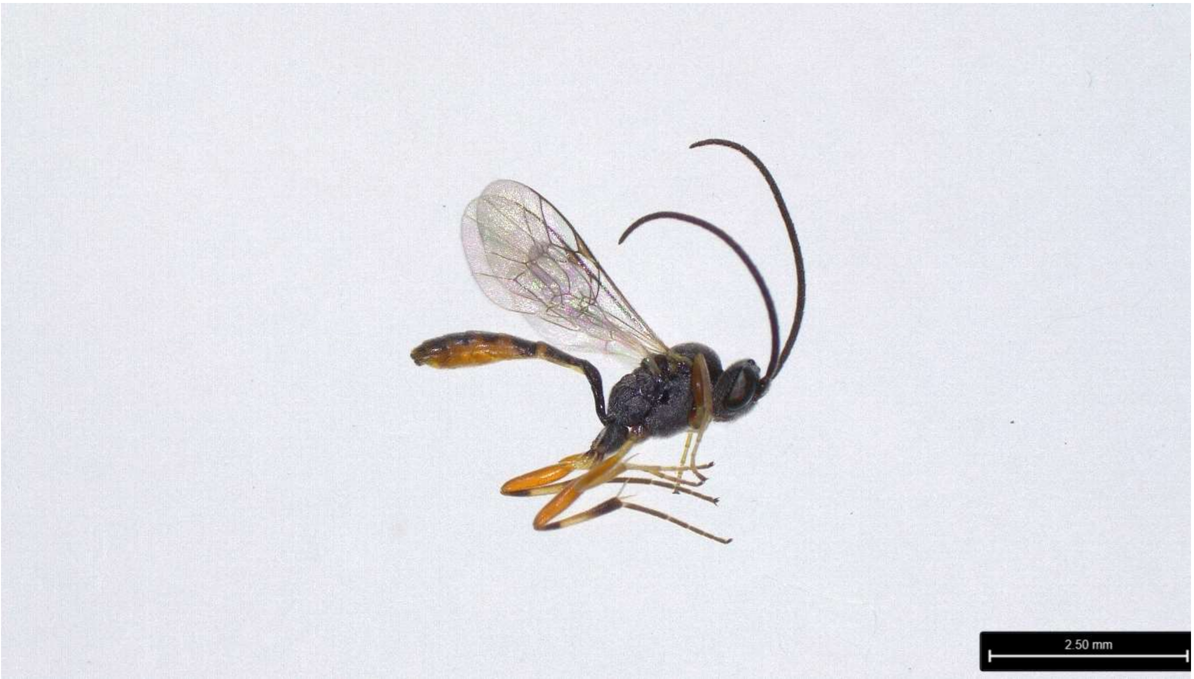

Supplementary Figure S7: Lateral view of *Campoletis chlorideae* Uchida

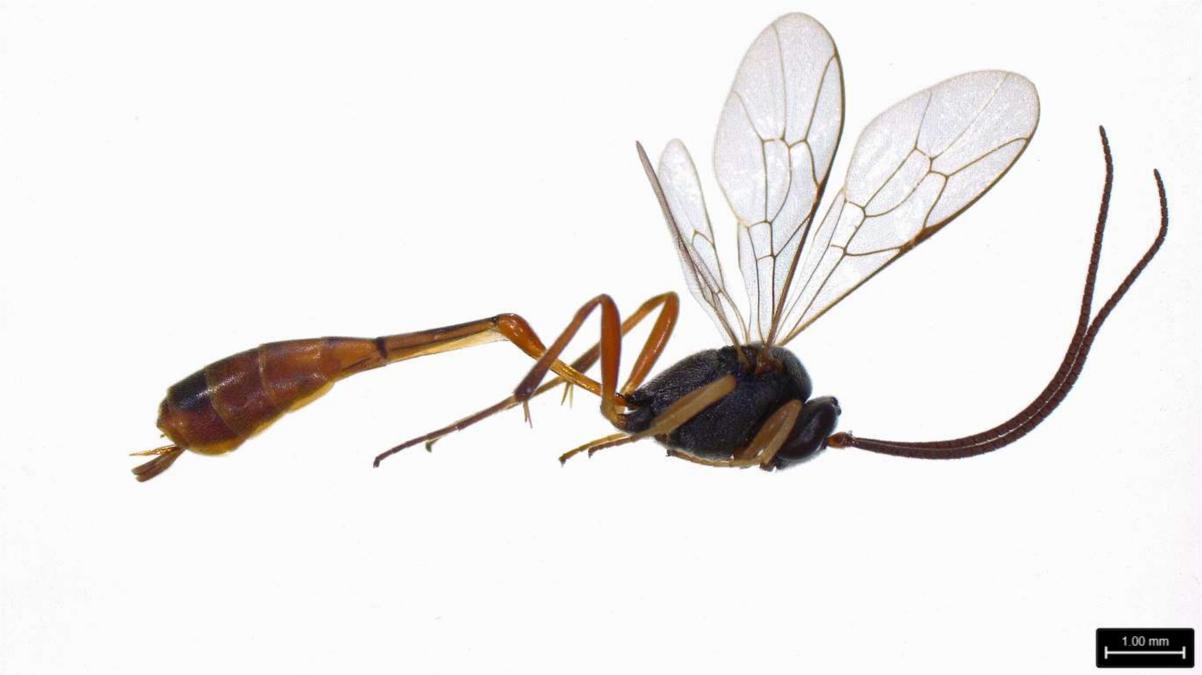

Supplementary Figure S8: Lateral view of *Charops bicolor* (Szepligeti)

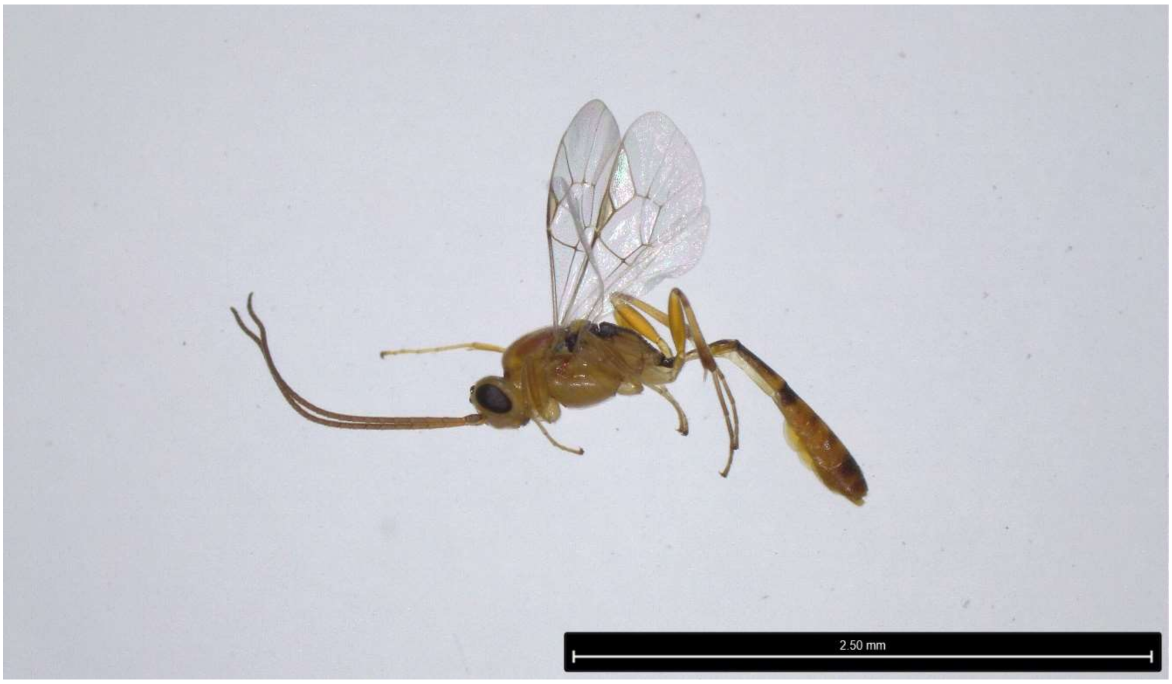

Supplemenatry Figure S9: Lateral view of *Temelucha* spp.

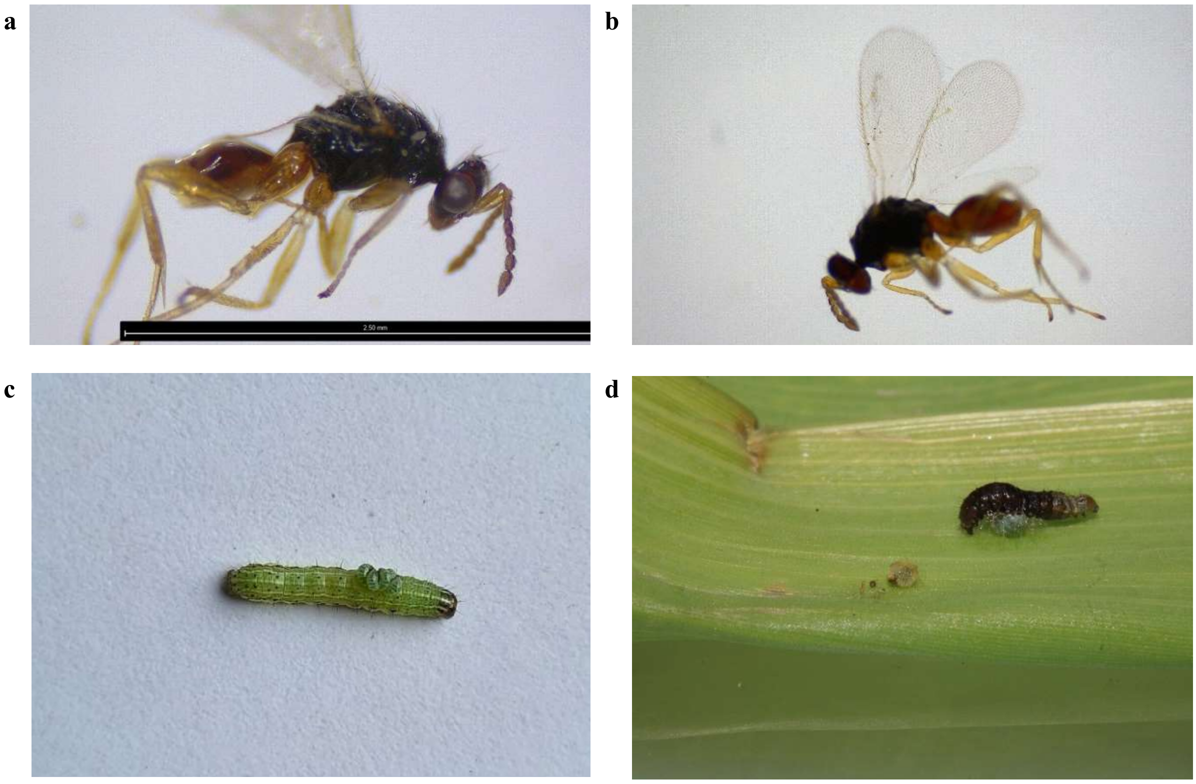

Supplementary Figure S10: *Euplectrus* spp. with lateral view (a), its wing region (b), attachement of parasitoid larvae to the dorsum of the host FAW caterpillar (c), cocoon to the underside of dead FAW host larva

Supplementary Table S6: FAW Parasitism rate across the survey districts in Indian Eastern Gangetic Plains

District	No. of Observations	Parasitism (%)	Exact level of significance for mean comparisons of all pairs by using Tukey HSD Test									
			Birbhum	Murshidabad	Malda	Dakshin Dinajpur	Uttar Dinajpur	Darjeeling	Coochbehar	Katihar	Purnea	Kisanganj
Birbhum, West Bengal	9	30.90 ± 2.55^{A} (0.0-59.09)	-	0.0004	<0.0001	<0.0001	<0.0001	<0.0001	<0.0001	<0.0001	<0.0001	0.063
Murshidabad, West Bengal	5	$10.97 \pm .42^{BC}$ (0.0-23.53)	0.0004	-	0.932	0.779	0.645	0.844	0.981	0.100	0.724	0.778
Malda, West Bengal	25	5.83 ± 1.50^{C} (0.0-10.0)	<0.0001	0.932	-	0.998	0.991	0.999	1.000	0.996	0.994	0.006
Dakshin Dinajpur, West Bengal	6	2.98 ± 3.12^{C} (0.0-7.41)	<0.0001	0.779	0.998	-	1.000	0.998	0.999	0.936	0.997	0.013
Uttar Dinajpur, West Bengal	14	3.43 ± 1.86^{C} (0.0-9.09)	<0.0001	0.645	0.991	1.000	-	1.000	0.997	0.856	1.000	0.001
Darjeeling, West Bengal	4	2.74 ± 3.83^{C} (0.0-7.41)	<0.0001	0.844	0.999	0.998	1.000	-	0.999	0.962	0.992	0.040
Coochbehar, West Bengal	9	6.19 ± 2.55^{BC} (0.0-16.67)	<0.0001	0.981	1.000	0.999	0.997	0.999	-	0.999	0.996	0.050
Katihar, Bihar	8	8.60 ± 2.70^{BC} (0.0-15.79)	<0.0001	0.100	0.996	0.936	0.856	0.962	0.999	-	0.903	0.258
Purnea, Bihar	6	2.56 ± 3.12^{C} (0.0-6.06)	<0.0001	0.724	0.994	0.997	1.000	0.992	0.996	0.903	-	0.001
Kishanganj, Bihar	7	18.70 ± 2.89^{AB} (0.0-38.89)	0.063	0.778	0.006	0.013	0.001	0.040	0.50	0.258	0.001	-
df		83										
F value		12.28										
p value		<0.0001										

Parasitism rate expressed as mean ± SE with a range in the parentheses; Districts not connected by the same letter are significantly different

Supplementary Table S7: Impact of insecticide application on FAW parasitism rate at different phenological growth stages of maize in Indian Eastern Gangetic Plains

Maize growth stage and insecticide presence	No. of Observations	Parasitism (%)	Exact level of significance for mean comparisons of all pairs by using Tukey HSD Test			
			Early Whorl with Insecticide	Early Whorl without Insecticide	Late Whorl with Insecticide	Late Whorl without Insecticide
Early whorl with insecticide	24	4.20 ± 2.05^{B} (0.0-21.43)	-	0.007	0.999	0.180
Early whorl without insecticide	38	12.90 ± 1.63^{A} (0.0-59.09)	0.007	-	0.012	0.959
Late whorl with insecticide	18	3.89 ± 2.37^{B} (0.0-13.33)	0.999	0.012	-	0.188
Late whorl without insecticide	13	11.28 ± 2.79^{AB} (3.03-33.33)	0.180	0.959	0.188	-
df		89				
F value		5.47				
p value		0.0017				

Parasitism rate expressed as mean ± SE with the range in the parentheses; Crop growth stages and insecticide presence not connected by the same letter are significantly different
